# Investigation of an outbreak of Shigella sonnei blaCTX-M-3 among men in England, 2025–26, using long- and short-read whole-genome sequencing

**DOI:** 10.1099/mgen.0.001817

**Published:** 2026-08-14

**Authors:** Jake D. Turnbull, David R. Greig, Clare R. Barker, Satheesh Nair, Katherine Lock, Craig Swift, Marie A. Chattaway, Kate S. Baker, Claire Jenkins

**Affiliations:** 1 Gastrointestinal Bacteria Reference Unit, UK Health Security Agency, London, NW9 5HT, UK; 2 NIHR Health Protection Research Unit in Gastrointestinal Infections, Universities of East Anglia & Newcastle, Norwich, UK; 3 NIHR Health Protection Research Unit in Public Health Genomics, University of Birmingham, Birmingham, UK; 4 Department of Genetics, University of Cambridge, Cambridge, CB2 3EH, UK

**Keywords:** long-read sequencing, multidrug resistance, outbreak, plasmids, *Shigella sonnei*, surveillance, third-generation cephalosporin

## Abstract

**Background.** Recent outbreaks of gastrointestinal symptoms among gay, bisexual and other men who have sex with men (GBMSM) have been caused by multidrug-resistant (MDR) *Shigella sonnei* carrying *bla*_CTX-M-27_ or *bla*_CTX-M-15_. To date, the *bla*_CTX-M-3_ variant has not been associated with sexually transmitted shigellosis in the UK.

**Methods.** Routine surveillance identified an outbreak *S. sonnei* among men in England in June 2025. Short-read sequencing data were analysed to identify antimicrobial resistance (AMR) determinants and to investigate the phylogenetic context of the outbreak. Long-read Oxford Nanopore Technology sequencing data were analysed to characterize the AMR-encoding plasmid content.

**Results.** Of the 83 cases linked to the outbreak cluster, 91.5% were adult males and none reported recent travel outside Europe; these outbreak characteristics (specifically adult male, no travel) are consistent with transmission among European GBMSM networks (Mitchell *et al*. 2019, Mitchell *et al*. 2021). Phylogenetic analysis placed the outbreak cluster within a wider clade historically associated with travel to the Middle East. The outbreak isolates were MDR; 90.4% (*n*=75/83) had *bla*_CTX-M-3_ located on an IncI1B/O plasmid. This is the first report of the IncI1B/O plasmid type and *bla*_CTX-M-3_ in GBMSM-associated MDR *S. sonnei* in England.

**Conclusions.** Acquisition of the IncI1B/O plasmid encoding *bla*_CTX-M-3_ represents an independent evolutionary event, separate from previously described GBMSM epidemics driven by acquisition of IncFII plasmids encoding *bla*_CTX-M-27_ or *bla*_CTX-M-15_. This study provides further evidence of the parallel emergence of *bla*_CTX-M_ variants conferring resistance to the third-generation cephalosporins. Whole-genome sequencing based surveillance and routine susceptibility testing of MDR *S. sonnei* are essential, as this pathogen continues to diversify and spread.

**Impact Statement**

This study describes an outbreak of *Shigella sonnei* among men notable for the acquisition of *bla*_CTX-M-3_. Using whole-genome sequencing, we showed that the outbreak strains did not emerge from *Shigella* lineages previously circulating within sexual networks of gay, bisexual and other men who have sex with men but instead represented a novel introduction from a clade previously associated with travel. *bla*_CTX-M-3_ was carried on an IncI1 B/O plasmid, distinct from the *bla*_CTX-M_ bearing IncFII plasmids identified in previous epidemics in England, highlighting the independent, parallel acquisition of resistance to the third-generation cephalosporins (3GC) in this setting. Our findings highlight the dynamic nature of plasmids encoding antimicrobial resistance (AMR) determinants circulating within tightly defined outbreak clusters. This has direct implications for treatment, as the rise of 3GC-resistant strains threatens the effectiveness of first and second line antibiotics, potentially increasing reliance on carbapenems. These results emphasize the importance of combining short and long read sequencing data for surveillance of mobile genetic elements in sexually transmitted gastrointestinal pathogens, to inform clinical management and targeted public health interventions aimed at controlling the spread of AMR.

## Data Summary

FASTQ-formatted whole-genome sequencing (WGS) reads (both Illumina and Oxford Nanopore Technologies; ONT) for all available isolates in this study can be found at the UKHSA NCBI BioProject (National Center for Biotechnology Information) project no. PRJNA315192. Further details can be found in Table S1, available in the online version of this article. The authors confirm all supporting data, code and protocols have been provided within the article or through supplementary data files.

## Introduction

Historically in the UK, the gastrointestinal pathogen *Shigella sonnei* was a common cause of travellers’ diarrhoea and community-acquired infectious intestinal disease. In 2009, notifications of shigellosis dramatically increased among gay, bisexual and other men who have sex with men (GBMSM) [1–3]. Since 2011, sexula transmission of *S. sonnei* has been endemic in the MSM community in England, acquiring resistance to azithromycin, fluoroquinolones and third-generation cephalosporins (3GC) [4–7].

Plasmid analysis of the first sustained shigellosis outbreak (*Shigella flexneri* 3 a in 2012) among GBMSM revealed that the multidrug-resistant (MDR) phenotype was explained by the presence of an IncFII plasmid designated pKSR100 (LN624486.1) carrying various combinations of the antimicrobial resistance (AMR) determinants *bla*_TEM-1_*, dfrA*17*, dfrA*1*, sul*1*, aadA*5*, ermB* and *mphA* [8]. Subsequently, pKSR100 spread globally and plasmids closely related to pKSR100 (>95% nucleotide identity) were identified in *S. flexneri* 2 a and *S. sonnei* [4]. More recently, outbreaks of sexually transmissible extensively drug-resistant (XDR) *S. sonnei* harbouring pKSR100-like plasmids carrying *bla*_CTX-M-27_ and *bla*_CTX-M-15_ have been described in the UK [6, 7, 9]. There is increasing evidence of the proliferation of XDR *S. sonnei*, and the ongoing evolution and dissemination of MDR and XDR plasmids among *Shigella* species on a global scale [10–16]. Against this backdrop, we conducted genomic analysis of an outbreak of *S. sonnei* harbouring *bla*_CTX-M-3_, a *bla*_CTX-M_ variant not previously associated with outbreaks of shigellosis among GBMSM in England.

## Methods

### Genomic DNA extraction, library preparation and illumina sequencing

Genomic DNA was extracted via a Qiagen Qiasymphony, library preparation using Nextera XT and Nextera Flex kits followed by sequencing on Illumina HiSeq 2500 and NextSeq 1000 platforms. Post-WGS, multi-locus sequence typing was performed using a modified version of Metric-Oriented Sequence Typer (https://github.com/phe-bioinformatics/MOST) [17]. AMR gene detection was performed using an in-house bioinformatics pipeline, GeneFinder (https://github.com/phe-bioinformatics/gene_finder) at a detection threshold of ≥91%. GeneFinder also detects resistance determinants that are mutationally acquired. Variant calling was performed by aligning sample reads (FASTQ) to the CC152 Ss046 reference genome (NC_007384.1) using BWA v0.7.3 [18], Samtools v0.7.17 [19] and GATK v2.6.5 UnifiedGenotyper [20] before import into SnapperDB v0.3.0 [21]. SNP addresses are generated as part of SnapperDB v 0.3.0 in for all isolates submitted to GBRU and are used to identify closely related strains and subsequently clusters. As described previously, SNP addresses use a pairwise clustering approach to assign distance threshold levels that descend: Δ250, Δ100, Δ50, Δ25, Δ10, Δ5, Δ0 [22]. Each isolate within one level is no more than that many SNPs apart from the next isolate within the same level. For this study, clusters were defined as three or more isolates that differ in the five SNP level. Lineage assignment was performed using Mykrobe v0.13.0 [23] using the *S. sonnei* scheme (20210201) results can be found in Table S1.

### Genomic DNA extraction, library preparation and Nanopore sequencing

High-molecular weight genomic DNA was extracted using the Fire Monkey HMW DNA extraction kit (Revolugen), each DNA sample was barcoded using the rapid barcoding kit (SQK-RBK014-24). Sequencing was performed on a FLO-MIN106 flow cell and on either a MinION Mk1C or GridION platform (ONT) for 48 h.

Base calling of raw FAST5 data was performed using Dorado v7.11.12 HAC model.

Downstream bioinformatics processing was completed using Cóimeáil v1.0 (https://github.com/ukhsa-collaboration/coimeail). Briefly, read trimming, filtering and assembly were performed using Porechop v0.2.4 (https://github.com/rrwick/Porechop), Filtlong v0.2.0 (https://github.com/rrwick/Filtlong) and Flye v2.9 [24], respectively. Assemblies were corrected using Racon v1.4.20 [25], Medaka v1.0.3 (ONT) using Nanopore reads, followed by Pilon v1.23 [26] and Racon v1.4.20. As the chromosome and plasmids from each assembly were circularized and closed, they were re-orientated to start with either *dnaA* or *repA* using Dnaapler version 1.3.0 [27].

### Plasmid analyses

Assemblies from Nanopore-sequenced isolates were screened for contigs of plasmid origin using the mob-typer component of MOB-suite v3.1.9 [28]. Contigs that were positive for a plasmid replication initiation gene (*rep*) gene were circularized, lacked 16S rRNA genes and were within a 0.06 Mash distance of a plasmid within the MOB-suite reference database were identified as plasmids. Mob-typer determined the *rep* type and assigned a 0.06 Mash cluster code to each plasmid. Plasmid contigs were extracted from WGS assemblies using seqkit v.2.11 [29] by using the fasta headers of contigs identified as plasmids; *seqkit grep -n -f plasmid_identifiers.txt all_assemblies_combined.fasta -o plasmid_contigs.fasta*.

Pairwise Mash distance between all plasmids was calculated using Mash version 2.3 dist [30]. AMR gene detection was carried out using ABRicate v1.0.1 (https://github.com/tseemann/abricate) with the NCBI AMRFinderPlus database downloaded on 27 November 2025. Note that, unlike GeneFinder, which was used to detect AMR determinants from the short read data, ABRicate only detects complete/acquired resistance genes and not AMR determinants that are mutationally acquired. GeneFinder is not currently compatible with long reads. Annotation was performed using Bakta software v1.9.4 with full database v5.1 [31]. Gene cluster visualization care was performed using clinker v0.0.32 with the commands, with identity threshold set at 0.85 [32].

Representative pKSR100 (LN624486.1, CP090162.1) and pAPR100 (CP090161.1) plasmid sequences (*n*=3), the Nanopore-sequenced plasmids described by Lefèvre S, *et al*. [12] and CP049176.1 were also included in the further analysis of plasmids for contextualization. NCBI accession identifiers for these plasmids are listed in Table S1.

Plasmid networks were created using a pairwise Mash distance value matrix loaded into an iGraph object using igraph v2.2.1, subsequently interacted with using tidygraph v1.3.1. The plasmid network was visualized using ggraph v2.2.2. Other visualizations were carried out using ggplot2 v4.0.1.

The networks are undirected and unweighted. Edges were plotted between plasmids/nodes pairs within 0.06 Mash distance of each other. A Mash distance of 0.06 was used as this results in highly homogeneous clusters when conducting complete linkage clustering. A Mash distance of 0.06 is set as the default primary clustering threshold within the mob-typer component of MOB-suite for this reason. Clustering here is performed as a means of grouping similar plasmids [33].

### Phylogenetic tree construction

Two phylogenies were constructed using the same methods, one at the t50 : 1 level (*n*=3,268) and one at the t25 : 38 level (*n*=131). For each phylogeny, a soft-core genome alignment of each cluster level was generated from SnapperDB v0.3.0. The Ss046 reference genome (NC_007384.1) was used to generate SNP addresses, although the reference genome is not included within the alignment or visualization of phylogenies. These alignments had recombination masked by Gubbins v2.4.1 using default parameters [34] and a maximum-likelihood phylogeny was constructed using IQTree v2.0.7 with the GTR+F+G4 model [35]. The t50 : 1 phylogeny was viewed in ITOL [36] and rooted on a sample most closely related to but outside this cluster.

## The study

### Surveillance of *S. sonnei* using whole-genome sequencing

**Fig. 1.**
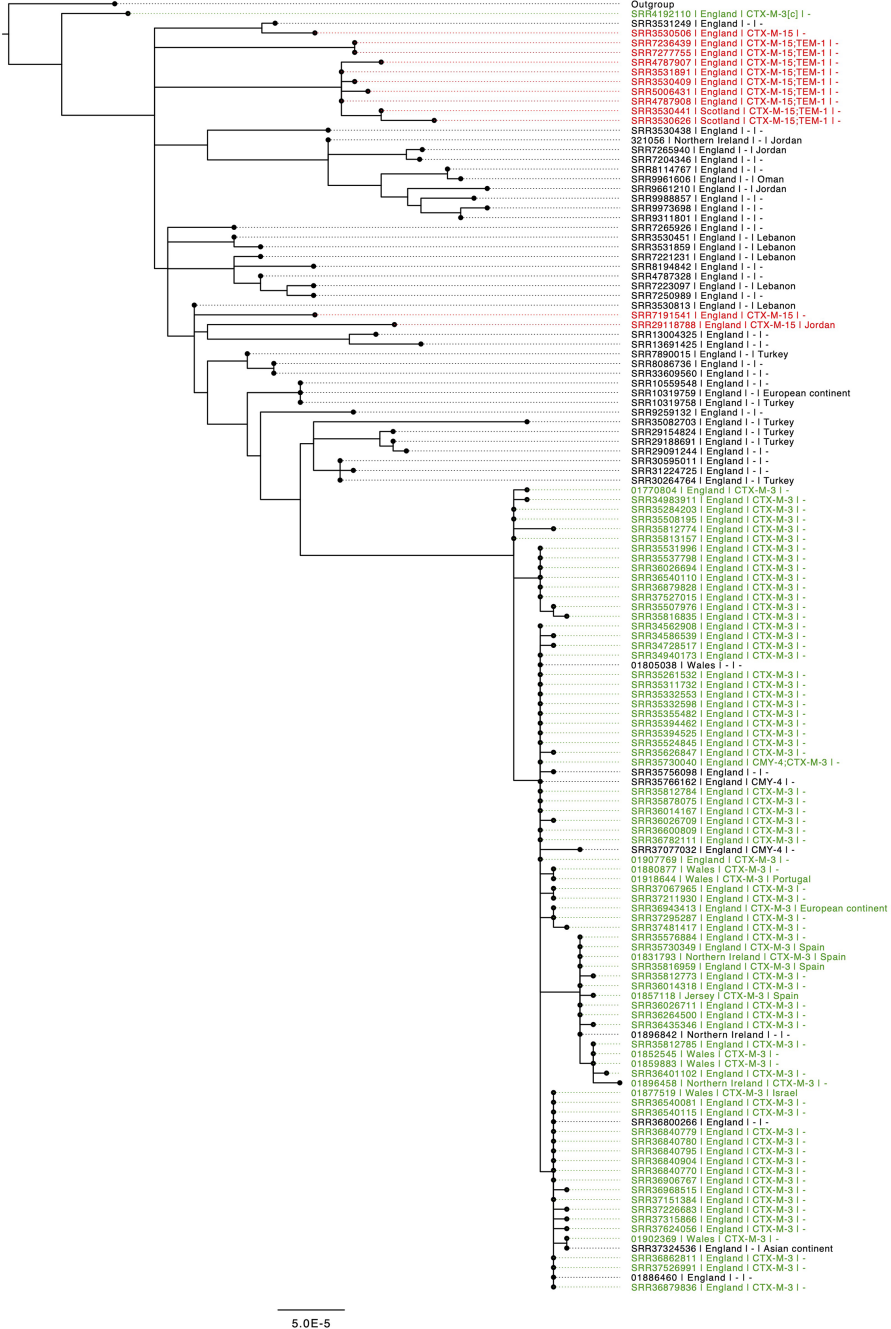
Maximum-likelihood phylogenetic tree of *S. sonnei* CC152 t25 : 38 partial clade (Lineage 3.6.1) of 132 UK genomes (alignment length 1,382 bp). The green coloured clade denotes t5 : 3811 cluster. Samples are coloured by the presence and type of *bla*_CTX-M_ subtype.

**Fig. 2.**
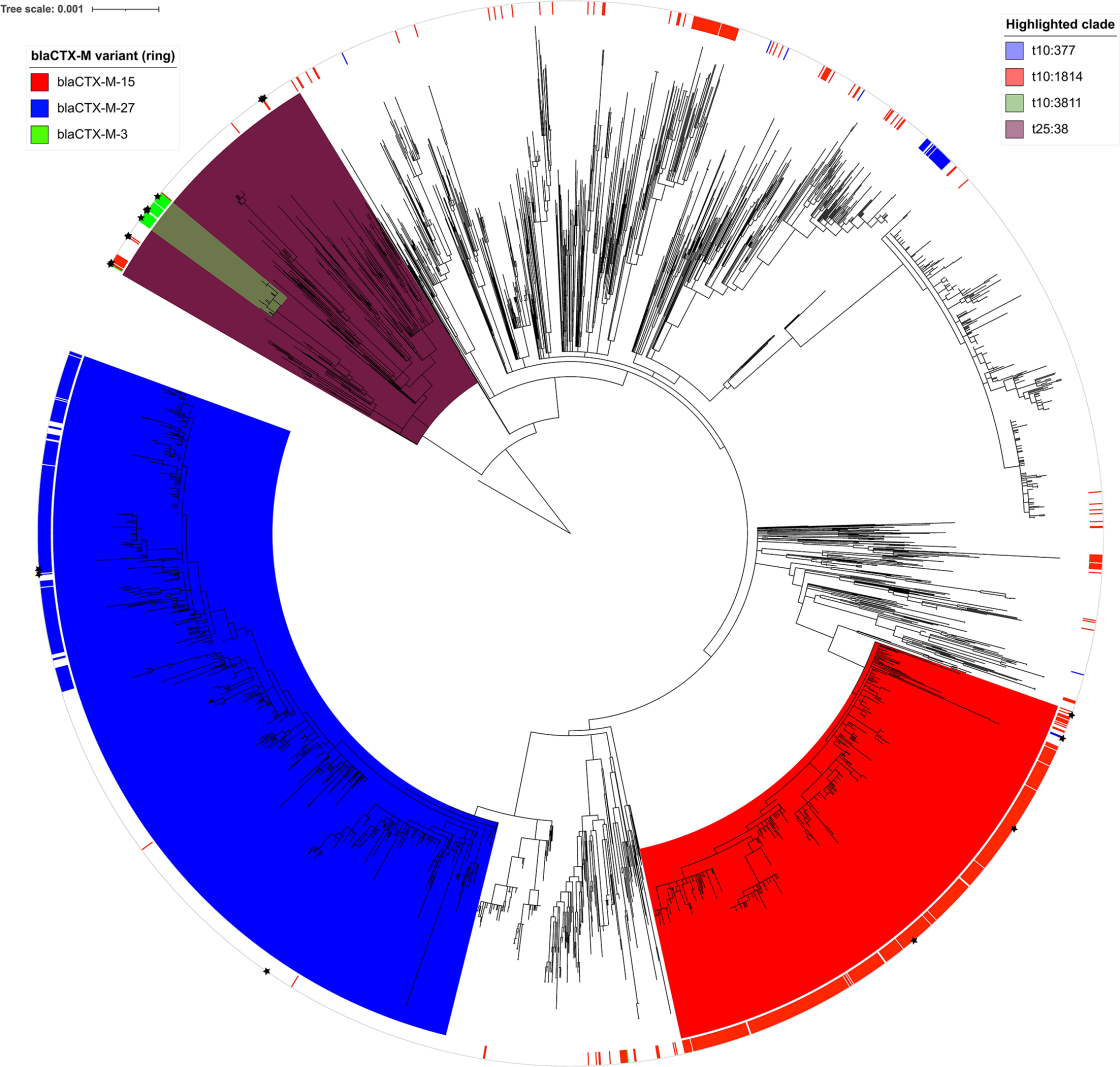
Maximum-likelihood phylogenetic tree of *S. sonnei* CC152 t50 : 1 (Lineage 3.6.1) of 3268 UK genomes (alignment length 13,197 bp). Clades are coloured by single-linkage clusters (SLCs) of interest, Green=t5 : 3811, Mauve=t25 : 38, Red=t10 : 1814 and Blue=t10 : 377. The outer ring is coloured by the presence of a CTX-M subtype, Red = *bla*_CTX-M-15_, Blue = *bla*_CTX-M-27_ and Green = *bla*_CTX-M-3_. Finally, the 16 samples labelled with a black star are those that underwent long-read sequencing.

UKHSA laboratory surveillance systems capture short-read WGS data of *S. sonnei* isolates referred from hospital laboratories (∼70% of the total isolated) [21, 37]. Genome-derived AMR profiling and SNP clustering were performed, as previously described [21, 38]. Additionally, 16 isolates (11 from this study and 5 from previous studies) were sequenced using an ONT MK1C MinION to elucidate and compare plasmid content. Isolates from this study were selected for ONT sequencing based on their AMR gene profile (representative isolates from each AMR profile) and their relative location in the wider *S. sonnei* population (Figs 1 and 2).

### Description of the outbreak

In June 2025, following routine analysis of WGS data, UKHSA surveillance systems identified an outbreak of closely related isolates of *S. sonnei* falling within the same 5 SNP single-linkage cluster (SLC), designated t5 : 3811. By mid-March 2026, 83 cases were linked to the outbreak, of which 91.6% (76/83) were adult males, and none reported travelling outside Europe in the weeks prior to onset of symptoms (Table 1, Fig. 1 and Table S1). Domestic acquisition and the predominantly adult male demographic means are characteristics of outbreaks associated with transmission within GBMSM networks [39, 40].

**Table 1.** Characteristics of isolates in the t5.3811 outbreak cluster compared to the isolates in the wider, contextual t25.38 clade. The wider clade excludes the outbreak cluster

	t5.3811 outbreak cluster (*n*=83)		Wider t25.38 clade (*n*=190)	
Time frame	June 2025–March 2026		Jan 2012–March 2025	
	Number	Percentage	Number	Percentage
Males	76	91.6	91	47.9
Females	7	8.4	99	52.1
Children	0	0	13	6.8
*Travel outside Europe*				
Yes	0	0	78	41.1
No	35	42.2	19	10.0
Unknown	48	57.8	93	48.9
*AMR*				
*bla*_CTX-M-3_	75	90.4	0	0
b*la*_CTX-M-15_	0	0	13	6.8

All 83 isolates in the t5 : 3811 5 SNP SLC outbreak cluster were MDR harbouring AMR determinants known to confer resistance to tetracycline (*tetA*), sulphonamides (*sul*2), trimethoprim (*dfrA*1) and reduced susceptibility to fluoroquinolones (*gyrA*:S83L). Within the outbreak cluster, 75/83 (90.4%) isolates had the *bla*_CTX-M-3_ gene and two isolates had *bla*_CMY-4_.

### Phylogenetic analysis

Although located within the same 50 SNP SLC (designated t50 : 1) as the previously described 3GC resistant UK MSM clusters harbouring plasmid encoded *bla*_CTX-M-27_ (t10 : 377) or *bla*_CTX-M-15_ (t10 : 1814), the t5 : 3811 outbreak described here fell within a different 25 SNP SLC or clade (designated t25 : 38) (Fig. 2). Excluding the t5 : 3811 outbreak isolates, the t25 : 38 clade comprised an additional 190 isolates, of which 99/190 (52.1%) were from females and 13/190 (6.8%) were from children. Of the 97/190 cases where a travel history was available, 78/97 (80.4%) reported travel outside Europe, the majority to the Middle East or Latin America (Table 1). Like the outbreak cluster (t5 : 3811), most isolates in the wider t25 : 38 cluster had the AMR determinants *tetA*, *sul*2, *dfrA*1 and *gyrA*:S83L. Twenty isolates had genes known to confer resistance to β-lactam antibiotics, *bla*_TEM-1_ (*n*=20) and/or *bla*_CTX-M-15_ (*n*=13), but none of the isolates outside of the outbreak cluster had *bla*_CTX-M-3_.

### Strains selected for long-read sequencing

To explore the genomic architecture of the AMR gene content, 11 isolates were selected for ONT long read sequencing within the t5.3811 outbreak (*n*=5) and the wider t25.38 clade (*n*=6) (Table 2). Five ONT sequences from the two previous outbreaks of 3GC *S. sonnei* among GBMSM were included in the analysis (*bla*_CTX-M-27_ *n*=2; *bla*_CTX-M-15_ *n*=3) (Table 2) [6, 7].

**Table 2.** AMR profiles of isolates selected for long-read sequencing. The AMR profiles were derived from short-read sequencing using GeneFinder. AMR genes marked with ^†^ or ^Δ^ were confirmed to be carried by plasmids or the chromosome (respectively) by screening contigs assembled from ONT reads for acquired AMR genes using ABRicate. AMR determinants without ^†^ or ^Δ^ were detected from short-read data but not attributable to a replicon. *bla_CMY-4_* was detected as *bla_CMY-4_* from short-read sequencing, but as *bla_CMY-138_* using ABRicate as indicated in Fig. 3. Isolates marked with *, only the IncFIA, IncFIC plasmid from each isolate was available for AMR gene screening using ABRicate

Strain reference	Sex	Cluster	β-lactams	Aminoglycoside	Fluoroquinolone	Macrolide	Trimethoprim	Tetracycline	Sulphonamide
01838808	M	t5 : 3811	*bla_CTX-M-3_* ^†^	*aph(6)-Id*^†^; *aph(3'')-Ib*^†^	*gyrA*_S83L	–	*dfrA1* ^Δ^	*tet(A*)^†^	*sul2* ^†^
01832991	M	t5 : 3811	*bla_CMY-4_^^^*	*aph(6)-Id*^†^; *aadA5*^†^; *aph(3'')-Ib*^†^	*gyrA*_S83L	*mphA* ^†^	*dfrA1*^Δ^; *dfrA7*; *dfrA17*^†^	*tet(A*)^†^	*sul2*^†^; *sul1*^†^
01832990	F	t5 : 3811	–	*aph(6)-Id*^†^; *aph(3'')-Ib*^†^	*gyrA*_S83L	–	*dfrA1* ^Δ^	*tet(A*)^†^	*sul2* ^†^
01830048	M	t5 : 3811	*bla_CMY-4_*^^^; *bla_CTX-M-3_*^†^	*aph(6)-Id*^†^; *aadA5*^†^; *aph(3'')-Ib*^†^	*gyrA*_S83L	*mphA* ^†^	*dfrA1*^Δ^*; dfrA7*; *dfrA17*^†^	*tet(A*)^†^	*sul2*^†^; *sul1*^†^
01798716	M	t5 : 3811	*bla_CTX-M-3_* ^†^	*aph(6)-Id*; *aph(3'')-Ib*	*gyrA*_S83L	–	*dfrA1* ^Δ^	*tet(A*)	*sul2*
01480908	M	t25 : 38	–	*aph(6)-Id*^†^; *aph(3'')-Ib*^†^	*gyrA*_S83L	–	*dfrA1* ^Δ^	*tet(A*)^†^	*sul2* ^†^
627421	F	t25 : 38	*bla_CTX-M-8_*	*aph(6)-Id*; *aph(3'')-Ib*	*gyrA*_S83L	–	*dfrA1* ^Δ^	*tet(A*)	*sul2*
418213	M	t25 : 38	*bla_CTX-M-15_*^†^; *bla_TEM-1_*^†^	–	*gyrA*_S83L	–	*dfrA1* ^Δ^	–	–
392977	F	t25 : 38	*bla_CTX-M-15_* ^†^	*aph(6)-Id*^†^; *aph(3'')-Ib*^†^	*gyrA*_S83L; qnrS-1^†^	–	*dfrA1* ^Δ^	*tet(A*)^†^	*sul2* ^†^
176409	F	t25 : 38	–	*aph(6)-Id*^†^; *aph(3'')-Ib*^†^	*gyrA*_S83L	–	*dfrA1* ^Δ^	*tet(A*)^†^	*sul2* ^†^
199861	M	t25 : 38	*bla_CTX-M-3_*	*aph(6)-Id*; *aph(3'')-Ib*	*gyrA*_S83L	–	*dfrA1* ^Δ^	*tet(A*)	*sul2*
1527854*	M	t10 : 377	*bla_CTX-M-27_* ^†^	*aph(6)-Id*; *aph(3'')-Ib* ; *aadA-5*^†^	*gyrA*_S83L_D87G; *parC*_S80I; qnrB-19	*ermB*^†^; *mphA*^†^	*dfrA1*^Δ^; *dfrA17*^†^	*tet(A*)	*sul2*; *sul1*^†^
1502196*	M	t10 : 377	*bla_CTX-M-27_* ^†^	*aadA-5* ^†^	*gyrA*_S83L_D87G; *parC*_S80I; qnrB-19	*ermB*^†^; *mphA*^†^	*dfrA1*^Δ^; *dfrA17*^†^	–	*sul1* ^†^
1662387	M	t10 : 1814	*bla_CTX-M-15_* ^†^	*aadA-5* ^†^	*gyrA*_S83L_D87G; *parC*_S80I	*mphA* ^†^	*dfrA1*^†Δ^; dfrA7; *dfrA17*^†^	*tet(B*)^†^	*sul2*^†^; *sul1*^†^
01322428	M	t10 : 1814	*bla_CTX-M-15_* ^†^	*aadA-5* ^†^	*gyrA*_S83L_D87G; *parC*_S80I	*mphA* ^†^	*dfrA1*^†Δ^; dfrA7; *dfrA17*^†^	*tet(B*)^†^	*sul2*^†^; *sul1*^†^
1318166	M	t10 : 1814	*bla_CTX-M-15_* ^†^	*aadA-5* ^†^	*gyrA*_S83L_D87G; *parC*_S80I	*mphA* ^†^	*dfrA1*^Δ^; dfrA7; *dfrA17*^†^	*tet(B*)^†^	*sul2*^†^; *sul1*^†^

### Selection of additional plasmid sequences for comparative analysis

Two previously reported MSM outbreak-associated azithromycin resistance-encoding IncFII plasmids, pKSR100 (LN624486.1, CP090162.1) and pAPR100 (CP090161.1), were also included to contextualize this analysis, due to the association of these plasmids with t50 : 1 isolates and *bla*_CTX-M_ gene acquisition on their variants. Similarly, recently described plasmids from XDR *S. sonnei* [12] and the publicly available sequence of plasmid p7111-69 (CP049176.1) were also included in comparative analysis. NCBI accessions for these plasmids are provided in Table S2.

### Plasmid analysis

**Fig. 3.**
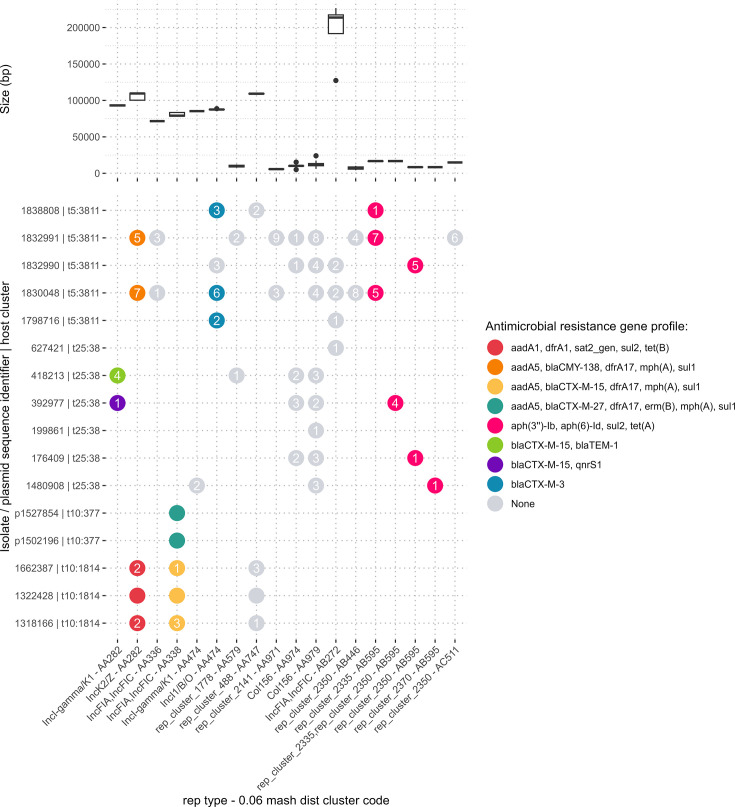
Distribution of the 54 identified plasmids across 16 long-read sequenced isolates. Each point on the graph represents a single plasmid contig. The number within each point is the contig number within each sample’s assembly e.g. the point that represents plasmid contig p01798716_2 will be labelled ‘2’. Plasmids are grouped based on their *rep* type and cluster code. The size distribution of each plasmid group in base-pairs is displayed. Isolates are grouped by SNP cluster with the t5 : 3811 outbreak isolates at the top of the second half of the figure, followed by t25 : 38, t10 : 377 and t10 : 1814 in descending order.

Within the 16 isolates selected for ONT long-read sequencing, 54 plasmids were identified, of which 16/54 were shown to carry AMR genes. To explore the relationships among these plasmids, clustering was undertaken and correlated to AMR gene content (Methods, Fig. 3). This revealed that the temporally and phylogenetically closely related t5 : 3811 outbreak strains had one or more of three AMR-encoding plasmids belonging to different *rep* types and 0.06 Mash distance clusters, and each encoding different AMR determinants (Fig. 3). These were:

Group 1: IncI1/B/O plasmids (87,005 bp–87,505 bp, plasmid cluster AA474) carrying *bla*_CTX-M-3_

Group 2: IncK2/Z plasmids (100,298 bp–100,299 bp, plasmid cluster AA282), carrying *aadA5*, *bla*_CMY-4_, *dfrA17*, *mphA* and *sul1*

Group 3: Plasmids typed as rep_cluster_2350/rep_cluster_2335 (8,422 bp – 16,762 bp, plasmid cluster AB595), carrying *aph3''-Ib, aph6-Id, sul2* and *tetA*

The plasmids in Group 3 (carrying *aph3''-Ib, aph6-Id, sul2* and *tetA*) were present in the isolates in both the t5 : 3811 outbreak cluster and the wider t25 : 38 clade (Fig. 3). However, the IncI1/B/O and IncK2/Z plasmids that were present in the outbreak cluster isolates were not detected in the isolates from the wider t25 : 38 clade (Fig. 3). This may suggest recent plasmid acquisition in this clade and/or that acquisition of the *bla*_CTX-M-3_ encoding plasmid was associated with transmission among GBMSM.

**Fig. 4.**
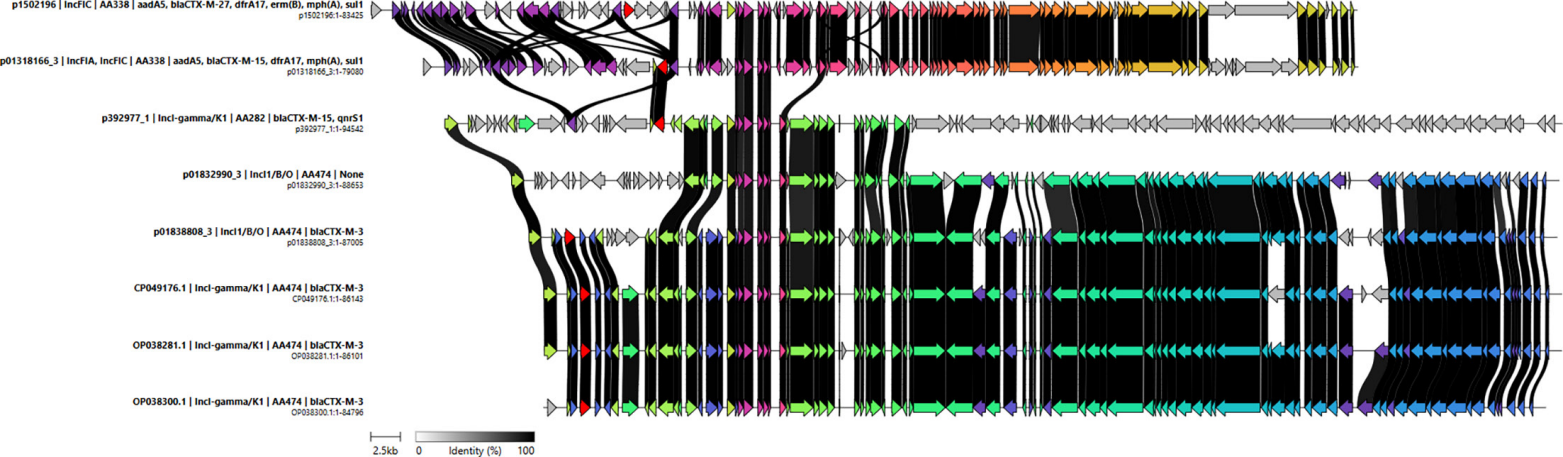
Clinker figure comparing exemplar plasmid sequences (top to bottom); a IncFIC *bla*_CTX-M-27_ positive plasmid from t10 : 377, IncFIA, IncFIC *bla*_CTX-M-15_ positive plasmid from t10 : 1814 (both from previous studies), a IncI-gamma/K1 *bla*_CTX-M-15_ positive plasmid from t25 : 38, a IncI1/B/O *bla*_CTX-M-3_ negative plasmid, a IncI1/B/O *bla*_CTX-M-3_ positive (Group 1) plasmid from the outbreak t5 : 3811 strain and reference plasmids CP049176.1, OP038281.1 and OP038300.1. Genes within 85% identity are grouped and coloured identically, with black lines connecting these groups between pairwise contig representations. The *bla*_CTX-M_ gene variants are coloured red.

Infrequently, t5 : 3811 isolates did not carry the *bla*_CTX-M-3_ gene. These isolates either carried a plasmid similar to the Group 1 plasmids, as in isolate 1832990, or not, as in isolate 1832991 (Fig. 3). This Group 1-like plasmid that carried *bla*_CTX-M-3_. A comparison of a Group 1 (p01838808_3) and Group 1-like (p01832990_3) plasmid is shown in Fig. 4. Where a t5 : 3811 isolate did not have *bla*_CTX-M-3_, these isolates may either be: (a) ancestral from a time prior to the acquisition of an entire Group 1 IncI/B/O plasmid, (b) carry the Group 1-like plasmid which itself may be ancestral to the Group 1 IncI/B/O plasmid, prior to the acquisition of *bla*_CTX-M-3_ or (c) have lost its Group 1 IncI/B/O plasmid. Isolate 199861 was positive for *bla*_CTX-M-3_ when examined for AMR genes from short-read data but negative when examined for AMR using ABRicate and assemblies derived from long reads. This is consistent with plasmid loss between the two sequencing runs.

The Group 1 IncI1/B/O plasmid was not present in the isolates associated with previous outbreaks of 3GC *S. sonnei* harbouring *bla*_CTX-M_ variants. Instead, both *bla*_CTX-M-27_ and *bla*_CTX-M-15_ determinants in these respective GBMSM outbreak strains were located on IncFIA/IncFIC plasmids (Fig. 3).

Two of the t5 : 3811 outbreak strains (01832991, 01830048) shared a similar IncK2/Z AA282 plasmid (within 0.06 Mash distances and the same *rep* type) with the three representative t10 : 1814 *bla*_CTX-M-15_ associated outbreak strains (01662387, 01322428 and 01318166), albeit encoding different AMR profiles with no AMR gene variants common between the two (Fig. 3).

**Fig. 5.**
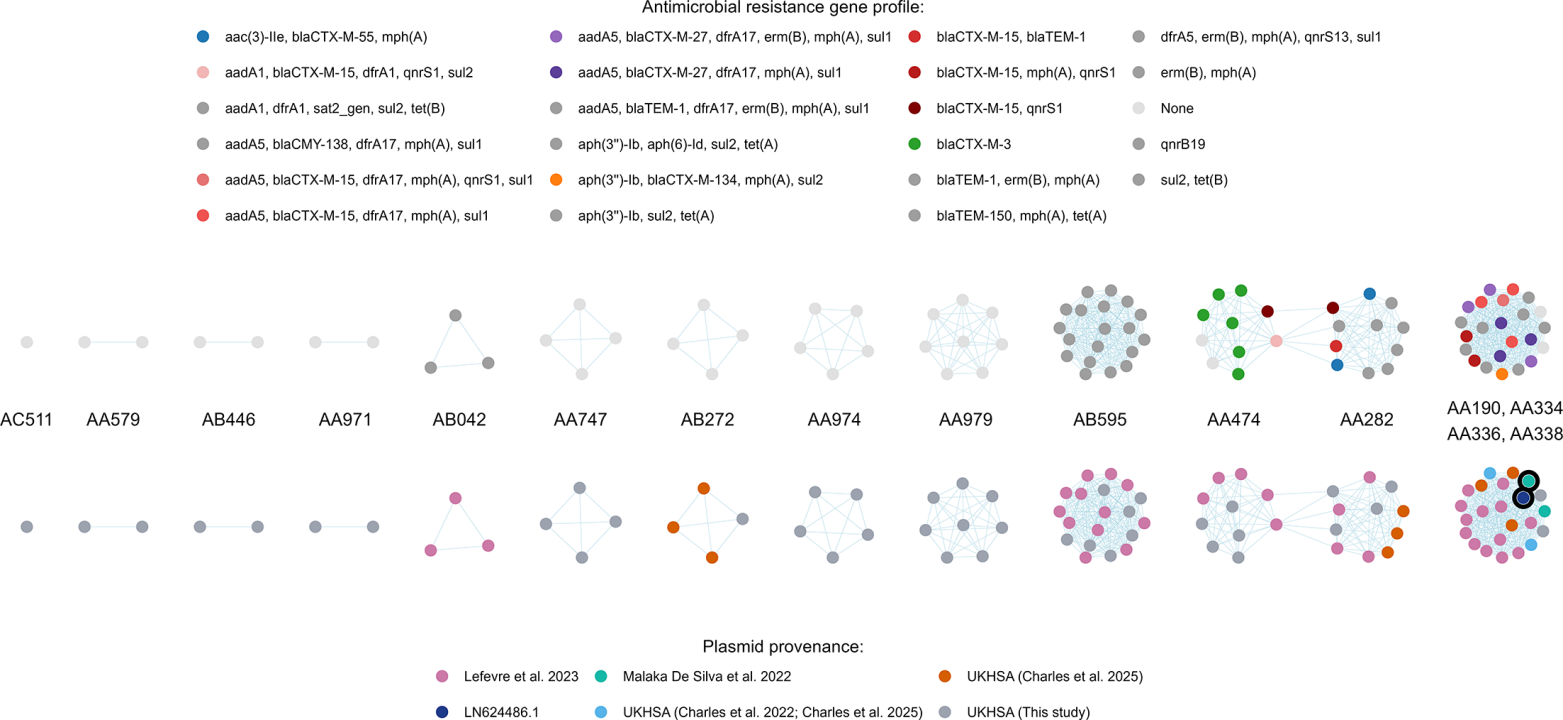
Network of 54 plasmids identified from UKHSA isolates and 40 reference plasmid sequences. Each node in the network represents a complete plasmid sequence and each edge between two nodes indicates that the sequences are within 0.06 Mash distance of one another. Both series of networks are identical except for how they are coloured. The top network series plasmid nodes are coloured, in shades, by plasmids containing *bla*_CTX-M_ variants, according to the inlaid key. Non-*bla*_CTX-M_ gene-containing plasmids carrying AMR genes are coloured in grey, and plasmids without detected AMR genes are shown in light grey. In the bottom network series, plasmid nodes are coloured by provenance. Two pKSR100 plasmids are encircled in black rings. The cluster codes pertaining to the plasmids in each network are annotated.

Comparison of the t5 : 3811 outbreak associated Group 1 IncI/B/O *bla_CTX-M-3_* positive AA474 cluster plasmids to publicly available plasmid sequences showed they shared similarity (<0.06 Mash distance) with the *bla*_CTX-M-3_ encoding IncI1 plasmids (CP049176.1, OP038281.1 and OP038300.1) and *bla_CTX-M-15_* encoding IncI1 plasmids (OP038275.1 and OP038292.1) previously described in France and Turkey [12] (Figs 4 and 5). The t5 : 3811 outbreak-associated Group 1 plasmids are also further demonstrated to be distinct from plasmids associated with prior outbreaks [6, 7], and pKSR100 and pAPR100 plasmids [41] (Fig. 5).

## Conclusions

The introduction of WGS for typing gastrointestinal pathogens greatly improved global surveillance of *S. sonnei* [23, 42]. Construction of the population structures and mapping of clades associated with travel and those associated with sexual transmission among GBMSM, enables us to track the acquisition of AMR and identify common exposures and risk factors that may contribute to emergence, transmission and persistence. In this study, phylogenetic analysis indicated that a novel t5.3811 outbreak strain evolved from a clade mainly associated with travel to the Middle East region or Latin America. Data from other studies have indicated that travel to regions where shigellosis is endemic may have a role in seeding domestic sexual transmission networks, and the deeper phylogenetic relationships described here may provide further evidence to support this hypothesis [7].

Short-read WGS has been used in this study to cluster strains, construct phylogenies and detect genes of interest, wheeras the selected application of long-read sequencing has enabled robust characterization of the plasmid content. The IncI1/B/O plasmid encoding *bla*_CTX-M-3_ was not detected in the historical isolates from the wider clade or in the isolates linked to the previous GBMSM epidemics of 3GC *S. sonnei*. Therefore, the outbreak described here is novel with respect to phylogeny, *bla*_CTX-M_ variant acquisition and plasmid type. Mash-based plasmid network analysis confirmed that the *bla*_CTX-M-3_ IncI1/B/O plasmid from the t5 : 3811 outbreak is closely related to *bla*_CTX-M-3_ and *bla*_CTX-M-15_ IncI plasmids from *S. sonnei* from France and Turkey. This study provides further evidence of the evolutionarily independent and parallel emergence of 3GC resistance determinants on different branches of the *S. sonnei* phylogeny.

We observed notable variation in the plasmid content between isolates within this small, temporally related 5 SNP SLC. Our analysis provides further insight into the dynamic nature of acquisition and loss of plasmids encoding resistance to 3GC antibiotics, as well as the change of AMR determinants within otherwise similar plasmids. As notifications of sexually transmissible shigellosis continue to rise and the circulating strains are increasingly resistant to 3GCs, we continue to highlight the need to create innovative solutions to reduce transmission in sexual networks where heavy antimicrobial use may be driving the emergence of MDR *S. sonnei* [42]. Enhanced surveillance, long and short read sequencing and antimicrobial susceptibility testing of *S. flexneri* and *S. sonnei* are all essential tools for monitoring the impact of antimicrobial usage and informing guidance and treatment regimens.

## Supplementary material

10.1099/mgen.0.001817 Supplementary Material 1.

## Abbreviations

AMR: antimicrobial resistance
dfrA1: trimethoprim
GBMSM: gay, bisexual and other men who have sex with men
3GC: third-generation cephalosporin
MDR: multidrug-resistant
ONT: Oxford Nanopore Technologies
SLC: single-linkage cluster
sul2: sulphonamides
tetA: tetracycline
WGS: whole-genome sequencing
XDR: extensively drug-resistant
